# CircRNA AFF4 promotes osteoblast cells proliferation and inhibits apoptosis via the Mir-7223-5p/PIK3R1 axis

**DOI:** 10.18632/aging.102524

**Published:** 2019-12-17

**Authors:** Bobin Mi, Yuan Xiong, Lang Chen, Chenchen Yan, Yori Endo, Yi Liu, Jing Liu, Liangcong Hu, Yiqiang Hu, Yun Sun, Faqi Cao, Wu Zhou, Guohui Liu

**Affiliations:** 1 Department of Orthopedics, Union Hospital, Tongji Medical College, Huazhong University of Science and Technology, Wuhan 430022, China; 2 Division of Plastic Surgery, Brigham and Women’s Hospital, Harvard Medical School, Boston, MA 02215, USA; 3 Department of Neurosurgery, Union Hospital, Tongji Medical College, Huazhong University of Science and Technology, Wuhan 430022, China

**Keywords:** circRNA, fracture healing, miRNA

## Abstract

Fracture healing is a complex process involving various cell types, cytokines, and mRNAs. Here, we report the roles of the circRNA AFF4/miR-7223-5p/PIK3R1 axis during fracture healing. We found that increased expression of PIK3R1 during fracture healing is directly associated with augmented proliferation and decreased apoptosis of MC3T3-E1 cells. Furthermore, miR-7223-5p targeted PI3KR1 and inhibited MC3T3-E1 proliferation while promoting apoptosis. CircRNA AFF4 acted as a sponge of miR-7223-5p, thereby promoting MC3T3-E1 cell proliferation and inhibiting apoptosis. Local injection of circRNA AFF4 into femoral fracture sites promoted fracture healing *in vivo* while the injection of miR-7223-5p delayed healing. These findings suggest that CircRNA AFF4 promotes fracture healing by targeting the miR-7223-5p/PIK3R1 axis, and suggests miR-7223-5p, CircRNA AFF4, and the miR-7223-5p/PIK3R1 axis are potential therapeutic targets for improving fracture healing.

## INTRODUCTION

Despite the advancement in medical technology, regeneration and restoration of bone defects remain challenging [1]. It is estimated that 10% of all the fractures result in non-union, which likely lead to a poor quality of life for the affected patients and an economical burden to society [2–4]. The fracture-healing process is highly complex, with an active involvement of various signaling molecules [4]. Under the regulation of these molecules, bones undergo remolding through either intramembranous ossification or endochondral ossification. Osteoblasts have been identified as key players that enhance bone repair [5]. Therefore, finding a way to promote osteoblast proliferation and suppress apoptosis during fracture healing could greatly improve fracture care.

miRNAs are small non-coding RNA molecules that act as post-transcriptional regulators of gene expression. By interacting with the 3’untranslated region of the target mRNAs, they regulate protein production, thereby modulating a series of physiological processes [6]. Circular RNAs (circRNAs) are a novel class of regulatory RNAs that are generated from precursor mRNA by back-splicing of exon(s). They can be located in the cytoplasm or the nucleus, and modulate gene expression either directly or indirectly, through regulation of miRNAs or RNA binding proteins, respectively [7]. Recently, cytoplasmic circRNAs have been well characterized as an endogenous “sponge” for miRNAs [8]. While circRNAs increase the expression of their target genes through inhibition of miRNAs, the expression of both of these types of RNA during fracture healing is poorly understood.

Recent microarray analyses have implicated many miRNAs in various diseases [9]. In the present study, we hypothesized that miRNA levels are altered during fracture healing compared to controls. To test this hypothesis, we analyzed publicly available data from the open-source gene expression omnibus (GEO) database. We indeed identified new miRNAs that are highly associated with fracture healing and predicted their downstream target genes using other open-source databases such as Targetscan (http://www.targetscan.org/mamm_31/) and miRDB (http://mirdb.org/). In addition, we investigated their upstream regulation by circRNAs using the CircBase (http://www.circbase.org), and explored their roles using in vitro and in vivo femoral fracture models. We successfully elucidated the circRNAs-miRNAs-mRNAs interactions during fracture healing.

## RESULTS

### miR-7223-5p inhibits fracture healing

**Figure 1:**
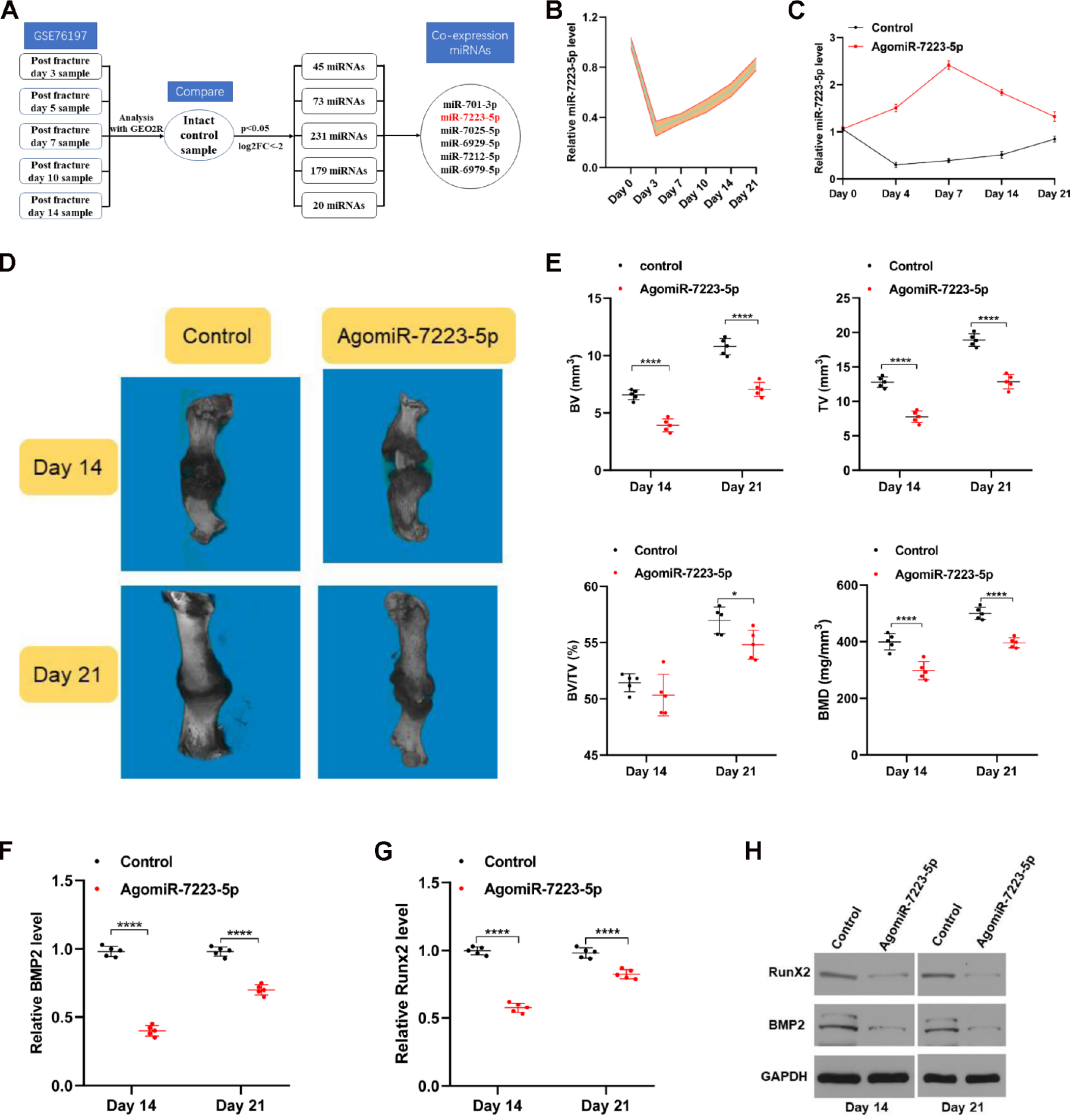
**miR-7223-5p was downregulated during fracture healing.** (**A**) Flow chart of downregulated miRNAs (log2 fold-change ≤ -2.0, *p*<0.05). (**B**) qRT-PCR analysis confirmed a decreased level of miR-7223-5p during fracture healing (n=5). (**C**) miR-7223-5p levels in calluses as measured by qRT-PCR (n=5). (**D**) MicroCT was used to reconstruct three-dimensional images of the fracture site. (**E**) Bone volume (BV), total volume (TV), and BV/TV were used to assess the calluses (n=5). (**F**–**H**) BMP2 and Runx2 levels in the callus samples from the mice on days 14 and 21 were measured by qRT-PCR and western blot (n=5).

We explored whether miRNAs are differentially expressed during fracture healing using data obtained from the Gene Expression Omnibus database (GSE76197). We found six miRNAs that were downregulated (fold change ≤-2.0, *p*<0.05) during fracture healing (Figure 1A), and selected miR-7223-5p for further analyses given its large changes in expression during fracture healing (Figure 1B). We will explore the effects of the other miRNAs during fracture healing in future studies.

To investigate the roles of miR-7223-5p during fracture healing, equal amounts of PBS (control) and agomiR-7223-5p were injected locally into fracture sites in mice and measured various parameters for each group during fraction healing. The levels of miR-7223-5p in the calluses were higher in the agomiR-7223-5p-treated group until Day 21 (Figure 1C). We performed micro computed tomography (microCT) and found that bone volume (BV), total volume (TV), and bone mineral density (BMD) were lower in the agomiR-7223-5p group compared to the control group, both on day 14 and day 21 following the fracture (Figure 1D, 1E). AgomiR-7223-5p treatment also inhibited the expression of some genes related with osteoblast differentiation (*e.g.*, BMP2 and Runx2) (Figure 1F–1H). Overall, the results of our *in vivo* study thus indicate that upregulation of miR-7223-5p delays fracture healing.

### miR-7223-5p suppresses MC3T3-E1 proliferation and promotes apoptosis

**Figure 2:**
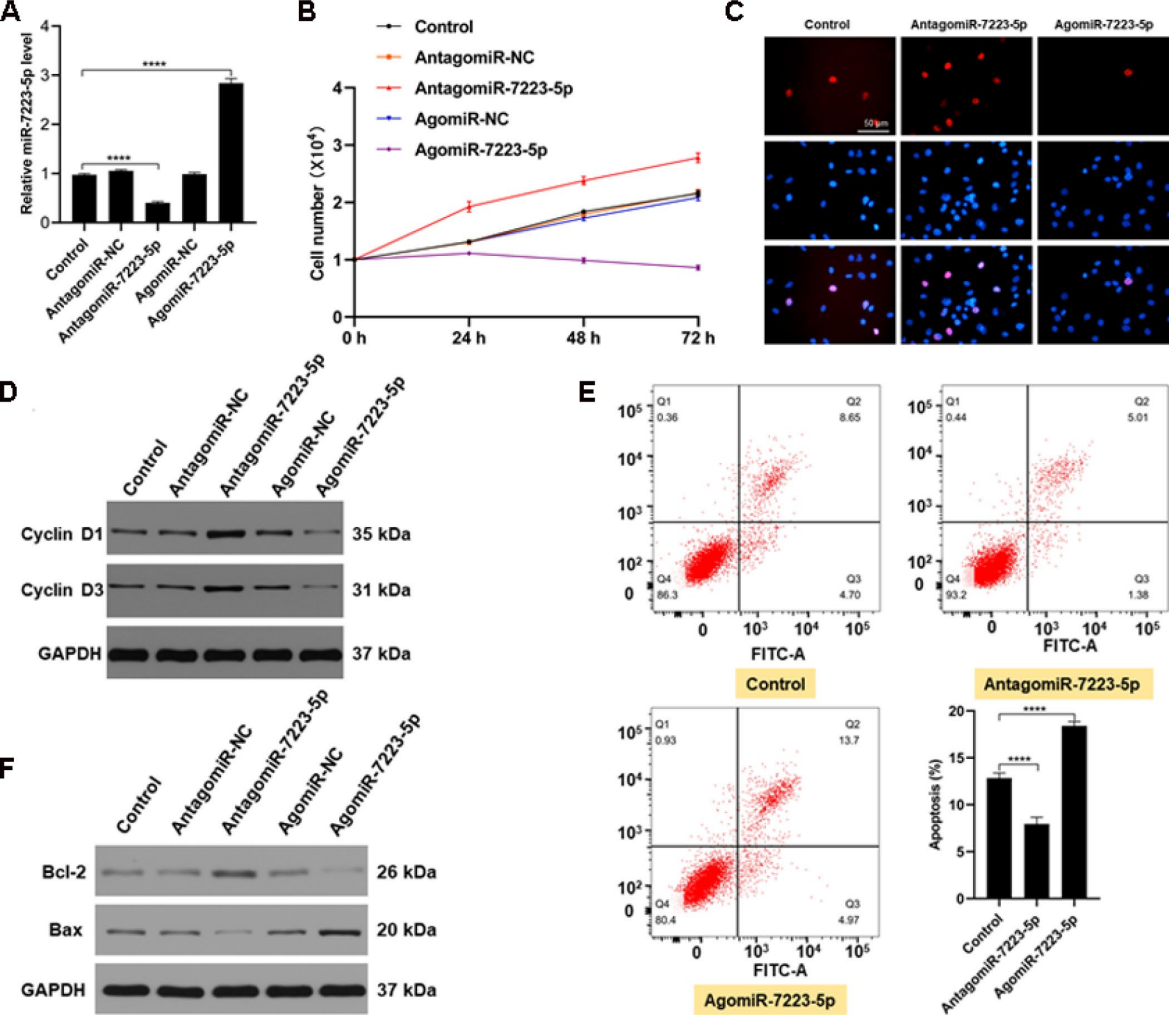
**miR-7223-5p suppressed the function of MC3T3-E1 cells *in vitro*.** (**A**) miR-7223-5p levels in MC3T3-E1 cells were measured by qRT-PCR after transfection with PBS (control), antagomiR-NC, antagomiR-7223-5p, agomiR-NC, or agomiR-7223-5p. (**B**, **C**) CCK8 and Edu assays were used to assess the proliferation of MC3T3-E1 cells after transfection with agomiR-7223-5p and antagomiR-7223-5p. (**D**) Cyclin D1 and cyclin D3 were detected by western blot after transfecting cells with agomiR-7223-5p or antagomiR-7223-5p. (**E**) The percentage of apoptotic MC3T3-E1 cells (Q2+Q3) was measured by flow cytometry 24 h after transfection with miR-7223-5p. Q1: dead cell; Q2: later apoptosis; Q3: early apoptosis; Q4: living cells. (**F**) Western blot showing Bax and Bcl-2 levels after transfecting cells with agomiR-7223-5p and antagomiR-7223-5p.

We investigated the role of miR-7223-5p during fracture healing *in vitro* using MC3T3-E1 cells. Following transfection with PBS, antagomiR-NC, antagomiR-7223-5p, agomiR-NC, or agomiR-7223-5p, we found elevated miR-7223-5p levels in the agomiR-7223-5p group, while the opposite effect was observed in the antagomiR-7223-5p group (Figure 2A). Results from CCK8 and Edu staining revealed lower cell numbers the agomiR-7223-5p-transfecred group (Figure 2B, 2C). Furthermore, Cyclin D1 and Cyclin D3, which promote progression through the cell cycle, were downregulated (Figure 2D) in the agomiR-7223-5p group compared to the control group, suggesting that the overexpression of miR-7223-5p inhibits MC3T3-E1 proliferation. We also found an increased number of apoptotic MC3T3-E1 cells in the agomiR-7223-5p group (Figure 2E). In agreement with such result, we also saw increased levels of the pro-apoptosis protein Bax and decreased levels of the anti-apoptosis protein Bcl-2 (Figure 2F). In contrast, when MC3T3-E1 cells were treated with antagomiR-7223-5p, they proliferated well and showed deceased apoptosis. Therefore, our *in vitro* results indicate that miR-7223-5p suppress MC3T3-E1 proliferation and promotes apoptosis.

### miR-7223-5p targets PIK3R1 and decreases MC3T3-E1 viability

**Figure 3:**
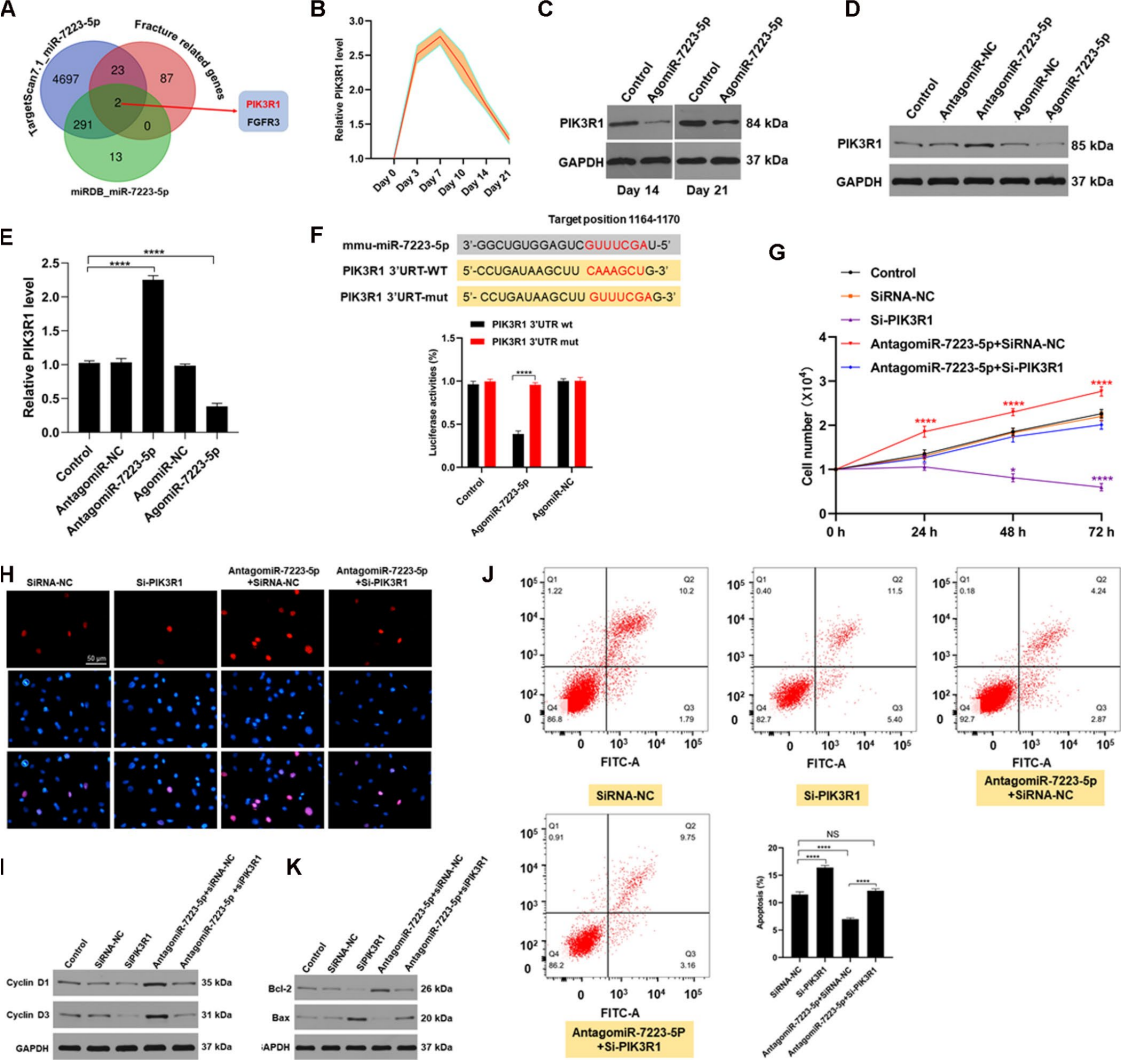
**miR-7223-5p regulates cells function via targeting FGFR3.** (**A**) Predicted targets of miR-7223-5p. (**B**) PIK3R1 levels in calluses during fracture healing measured by qRT-PCR. (**C**) PIK3R1 expression in calluses on days 14 and 21 post-fracture after PBS and agomiR-7223-5p injection. (**D**, **E**) PIK3R1 expression measured by western blot and qRT-PCR after transfecting cells with agomiR-7223-5p or antagomiR-7223-5p. (**F**) The predicted target site of miR-7223-5p on PIK3R1 and luciferase activities. (**G**, **H**) CCK8 and Edu staining showing MC3T3-E1 proliferation after transfection with siPIK3R1 or antagomiR-7223-5p+siPIK3R1. (**I**) Cyclin D1 and cyclin D3 levels in cells measured by Western blot. (**J**) Cell apoptosis (Q2+Q3) assessed by flow cytometry 24 h after transfection with si-PI3KR1. Q1: dead cell; Q2: later apoptosis; Q3: early apoptosis; Q4: living cells. (**K**) Apoptosis-related proteins were detected by Western blot.

To investigate the mechanism underlying miR-7223-5p’s suppression of MC3T3-E1 viability, we first predicted its downstream mRNA targets using bioinformatic tools. We found that PIK3R1 is a likely target of miR-7223-5p (Figure 3A). Indeed, PIK3R1 levels were elevated during the first several days of fracture healing *in vivo* (Figure 3B). After agomiR-7223-5p injection into the fracture site, PIK3R1 expression decreased (Figure 3C). Overexpression of miR-7223-5p suppressed PIK3R1 expression in MC3T3-E1 cells while miR-7223-5p knockdown increased it (Figure 3D, 3E). AgomiR-7223-5p treatment resulted in reduced luciferase signals from the PIK3R1 reporter in wild type (WT) cells, but had no effect on mutants (Figure 3F). PIK3R1 knockdown suppressed MC3T3-E1 proliferation and increased apoptosis, with both effects being rescued by miR-7223-5p inhibition (Figure 3G–3K), suggesting that miR-7223- targets PIK3R1.

### CircRNA AFF4 acts as sponge of miR-7223-5p

**Figure 4:**
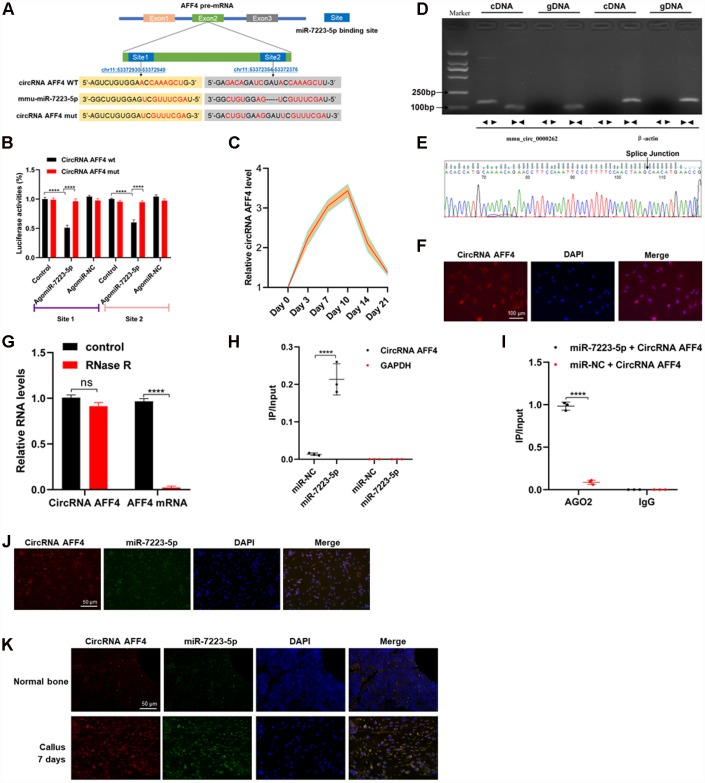
**CircRNA AFF4 acted as a sponge of miR-7223-5p.** (**A**) The target site of miR-7223-5p on circRNA AFF4 was predicted by CircBase. (**B**) Luciferase reporter activity of circRNA AFF4 with miR7223-5p. (**C**) circRNA AFF4 levels in the calluses measured by qRT-PCR during fracture healing. (**D**) Agarose gel electrophoresis showing that divergent primers amplified circRNAAFF4 in complementary DNA (cDAN) but not genomic DNA (gDNA). (**E**) The amplified product of specific divergent primers was confirmed to be of circRNA AFF4 by sequencing. (**F**) Expression and location of circRNA AFF4 in MC3T3-E1 determined by fluorescence *in situ* hybridization (FISH). circRNA AFF4 was labelled with Cy3. (**G**) circRNA AFF4 and AFF4 mRNA levels in MC3T3-E1 cells with or without RNase R treatment measured by qRT-PCR. (**H**) MC3T3-E1 cells were transfected with biotinylated miR-7223-5p or miR-NC. circRNA AFF4 and GAPDH levels quantified by qRT-PCR, and relative ratios of immunoprecipitate to input. (**I**) AGO2 RNA immunoprecipitation in MC3T3-E1 cells transfected with miR-7223-5p. circRNA AFF4 levels measured by qRT-PCR, and relative ratio of IP to input. (**J**) RNA FISH showing co-localization of circRNA AFF4 and miR-7223-5p in the cytoplasm of MC3T3-E1 cells. (**K**) RNA FISH showing co-localization of circRNA AFF4 and miR-7223-5p in the cytoplasm of cells derived from bone and calluses. CircRNA AFF4 and miR-7223-5p were labelled with Cy3 and AFM, respectively. The cell nuclei were stained with DAPI.

In order to identify the upstream regulators of miR-7223-5p, we used CircBase to predict the circRNAs that regulate miR-7223-5p. Our results showed that circRNA AFF4 binds to miR-7223-5p (Figure 4A). Luciferase reporter assay showed that WT circRNA AFF4 signals were suppressed by miR-7223-5p treatment while circRNA AFF4 mutations abolished the inhibitory effect of miR-7223-5p (Figure 4B). CircRNA AFF4 was markedly increased during the first several days of fracture healing (Figure 4C). The predicted splice junction of circRNA AFF4 was validated in MC3T3-E1 cells, and the product was amplified using divergent primers (Figure 4D, 4E). FISH analysis demonstrated that circRNA AFF4 was mainly located in the cytoplasm of MC3T3-E1 cells (Figure 4F). CircRNA AFF4 was more resistant to RNase R treatment compared to mRNA AFF4 (Figure 4G). Pull-down assays revealed a higher level of circRNA AFF4 in the miR-7223-5p-captured fraction compared to the miR-NC-capture fraction (Figure 4H). AGO2 immunoprecipitation showed that after the transfection of cells with miR-7223-5p, the endogenous circRNA AFF4 was pulled down by AGO2 (Figure 4I). RNA FISH showed co-localization of circRNA AFF4 and miR-7223-5p in the cytoplasm of MC3T3-E1 cells (Figure 4J) and the cells in the uninjured bone and calluses (Figure 4K). Taken together, our data indicate that circRNA AFF4 can directly bind to miR-7223-5p.

### CircRNA AFF4 promotes proliferation and inhibits apoptosis of MC3T3-E1 cells *in vitro*

**Figure 5:**
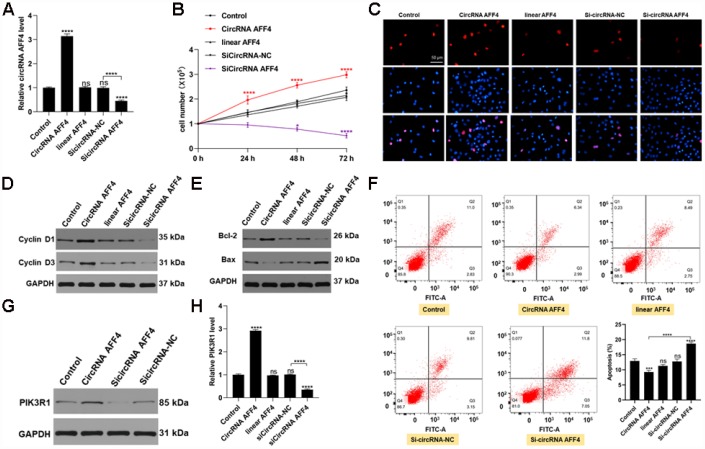
**CircRNA AFF4 functioned in MC3T3-E1 cells.** (**A**) circRNA AFF4 levels measured by qRT-PCR. (**B**, **C**) Effects of circRNA AFF4 on cell proliferation assessed by CCK8 and Edu staining. (**D**) Cyclin D1 and Cyclin D3 expression assessed by Western blot. (E) Bcl-2 and Bax expression measured by Western blot. (**F**) Flow cytometry was used to assess MC3T3-E1 apoptosis after transfection with circRNA AFF4. (**G**, **H**) PIK3R1 levels after transfection with circRNA AFF4 measured by western blot and qRT-PCR.

To further investigate the roles of circRNA AFF4 during fracture healing, we transfected MC3T3-E1 cells with circRNA AFF4 or si-circRNA AFF4. The transfection efficiency was assessed by qRT-PCR, and the result confirmed that circRNA AFF levels were increased in the circRNA AFF4-transfected cells (Figure 5A). CCK8 assay revealed that circRNA AFF promotes cell proliferation, a result that was further confirmed by Edu staining (Figure 5B, 5C). CircRNA AFF4 cells exhibited highly levels of Cyclin D1 and Cyclin D3, proteins that promote cell cycle progression (Figure 5D). Regard apoptosis, the pro-apoptotic protein Bax was suppressed while the anti- apoptotic protein (Bcl-2) was induced by circRNA AFF4 transfection (Figure 5E). Moreover, circRNA AFF4 decreased the percentage of apoptotic cells (Figure 5F). In addition, overexpression of circRNA AFF4 resulted in higher levels of PIK3R1 (Figure 5G, 5H). Collectively, these data indicated that circRNA AFF4 promotes MC3T3-E1 cell proliferation and inhibits apoptosis through targeting miR-7223-5p and PIK3R1.

### Therapeutic use of circRNA AFF4 in a mouse model of femoral fracture

**Figure 6:**
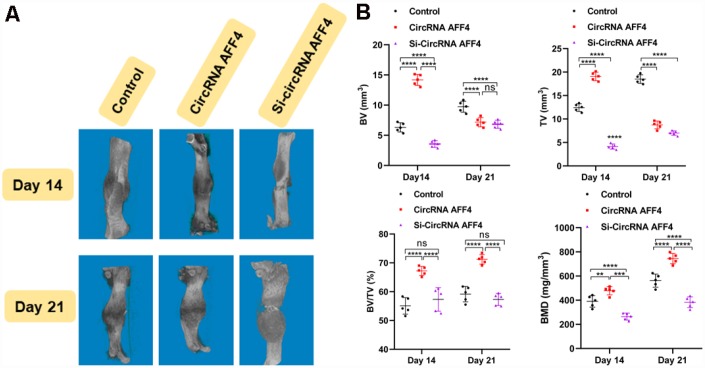
**Local injection of circRNA AFF4 promoted fracture healing *in vivo*.** (**A**) MicroCT was used to reconstruct three-dimensional images of fracture sites; (**B**) BV, TV, BV/TV, and BMD were used to assess the quality of calluses.

To explore the role of circRNA AFF4 in facture healing *in vivo*, we locally injected equal amounts of PBS, circRNA AFF4, and si-circRNA AFF4 into the fracture sites. Our microCT results showed that the BV, TV, and BMD of the calluses were increased by the injection of circRNA AFF4 (Figure 6A, 6B). Taken together, these results indicate a potential therapeutic effect of circRNA AFF4 in promoting fracture healing.

## DISCUSSION

Despite recent improvements in our knowledge of miRNAs and circRNAs, our understanding of their roles during fracture healing is still limited. Dysregulation of miRNAs has been widely reported in various biological processes, such as cell migration, cell proliferation, and cell metabolism, among others [10]. Several miRNAs have been reported to be involved in bone formation, exhibiting pro- or anti-osteogenesis effects during fracture healing [11, 12]. In order to further characterize the miRNAs that regulate fracture healing, we used microarray data to identify the miRNAs that are dysregulated during fracture healing and found that miR-7223-5p was downregulated in calluses during fracture healing compared normal bones. We confirmed that miR-7223-5p levels were downregulated in our *in vivo* fracture mouse model during the first several days of fracture healing and gradually increased with bone formation. In order to further verify the effects of miR-7223-5p on fracture healing, we injected agomiR-7223-5p into the fracture sites. Although we injected miR-7223-5p on days 0, 4, and 7, miR-7223-5p levels were still higher in the agomiR-7223-5p group than in the control group on day 21. Overall, our *in vivo* data showed that fracture healing was suppressed by injection of agomiR-7223-5p. Results from our *in vitro* analyses were consistent with those of from our *in vivo* experiments, showing that miR-7223-5p overexpression inhibited proliferation and promoted apoptosis of MC3T3-E1 cells.

PIK3R1 promotes the formation of phosphatidylinositol (3, 4, 5)-trisphosphate (PIP3) from phosphatidylinositol (4, 5)-bisphosphate (PIP2) in the plasma membrane, thereby activating AKT [13]. Previous studies reported that cell survival requires activation of the PI3K/AKT pathway [14, 15]. Consistent with previous studies that showed that PIK3R1 could be regulated by miRNAs [16], our study shows that PIK3R1 is a target gene of miR-7223-5p. Knockdown of PIK3R1 expression resulted in reduced cell proliferation and enhanced apoptosis miR-7223-5p silencing rescued the inhibitory effects on cellular functions induced by PIK3R1 knockdown alone, suggesting that miR-7223-5p inhibits proliferation and induces apoptosis via targeting PIK3R1.

CircRNAs can be generated from exons, intronic or intergenic regions. Their ability to regulate mRNA expression depends on their location within the cell. In the present study, we found that circRNA AFF4 was mainly located in the cytoplasm of MC3T3-E1 cells. Previous studies reported that the cytoplasmic circRNAs act as an endogenous miRNA sponge of miRNA, thereby affecting the functions of the target miRNAs at the post-transcriptional level [17]. The circular form of circRNAs protect them from degradation by exonucleases [18], thus allowing longer exertion of their effects in cells. We observed increased levels of circRNA AFF4 during the first several days following fracture *in vivo*. Its expression pattern was the opposite of that of miR-7223-5p, suggesting that circRNA AFF4 may promote fracture healing by suppressing miR-7223-5p. RIP, FISH, and luciferase assays *in vitro* revealed that miR-7223-5p was a downstream target of circRNA AFF4. CircRNA AFF4 promotes proliferation and inhibits apoptosis of MC3T3-E1 cells. In addition, PI3KR1 expression was enhanced by circRNA AFF4. Overall, our results indicate that circRNA AFFT, miR-7223-5p, and PI3KR1 compose an axis that regulates fracture healing.

Previous studies reported that apoptosis and senescence may occur after RNA transfection into cells [19], both of which inhibit cell proliferation and migration. However, it normally takes a longer time (more than 48 h) to trigger cell senescence than apoptosis [20, 21]. In the present study, we measured cell proliferation and apoptosis 24 h after miR-7223-5p and circRNA AFF4 transfection. Impaired proliferation of MC3T3-E1 cells may have been the main contributing factor inducing cell apoptosis.

The results from our *in vitro* and *in vivo* experiments using an animal model warrant further studies with calluses collected from patients. In addition, fracture healing is highly complex; therefore, there may exist other cell types (preosteoblasts, osteoblasts, fibroblasts, stromal cells, stem cells, etc.), non-coding RNAs, and mRNAs involved in fracture healing. Further studies are needed to understand the roles of circRNAs, miRNAs, and mRNAs on various cell types during fracture healing.

**Figure 7:**
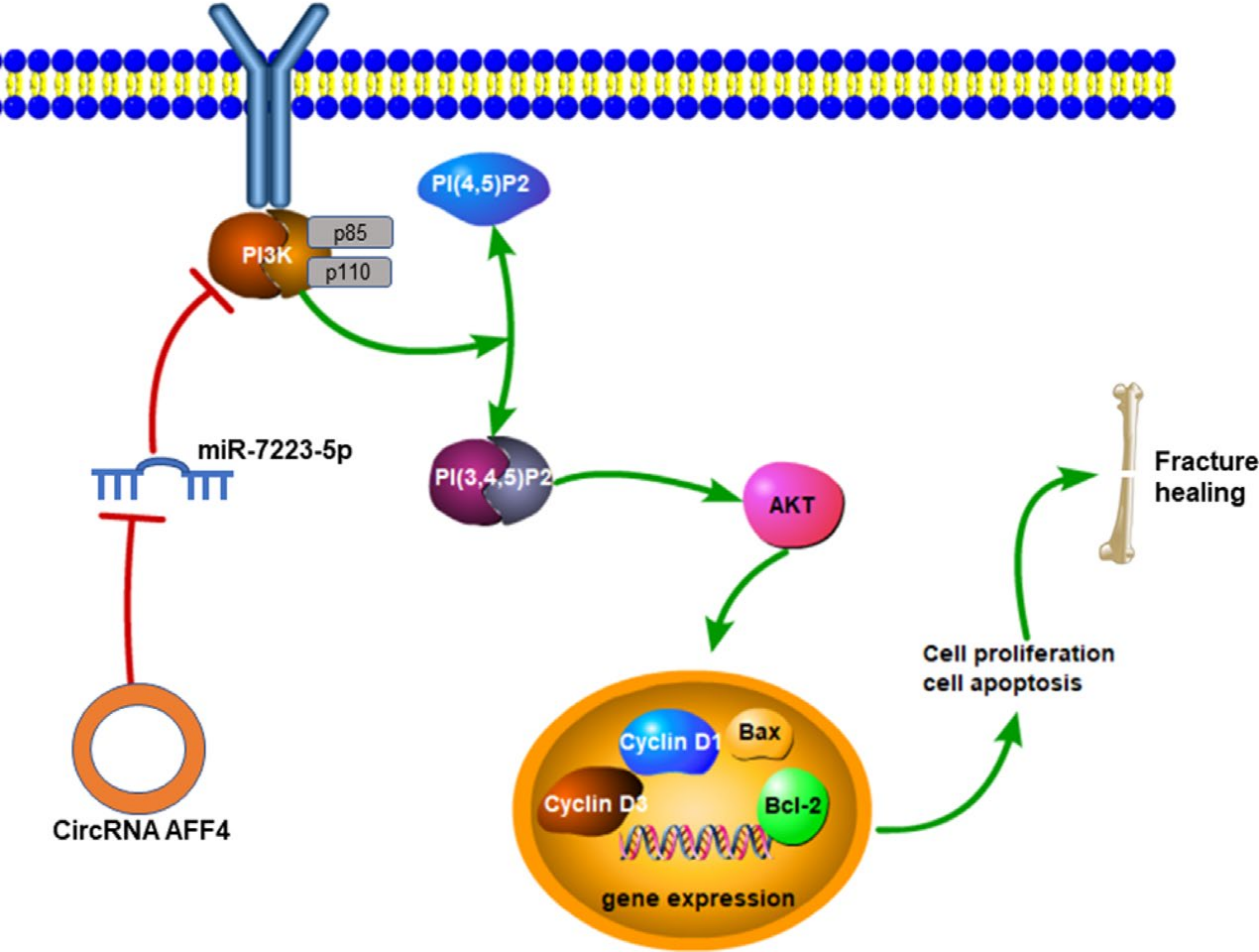
**Mechanisms of circRNA AFF4 mediated fracture healing.** CircRNA AFF4 acts as sponge of miR-7223-5p, leading to PI3K-AKT activation, which accelerates fracture healing via promoting cell proliferation and inhibiting apoptosis.

In conclusion, our study showed that circRNA AFF4 could promote proliferation and inhibit apoptosis of MC3T3-E1 cells through the miR-7223-5p/PIK3R1 axis (Figure 7), which may be exploited for therapeutic benefits to improve fracture healing.

## MATERIALS AND METHODS

### Cell culture and transfections

MC3T3-E1 cells were cultured in α-modified Eagle’s medium (α-MEM) with 10% fetal bovine serum in 5% C02 at 37 °C. The miRNAs, siRNAs and plasmid circRNAs were constructed by GenePharma (Shanghai, China). Cells were transfected with the RNAs using lipofectamine 3000 (Invitrogen, USA). The transfection rates of agomiR-7223-5p and antagomiR-7223-5p, circRNA AFF4, linear AFF4, and siRNA PIK3R1 were evaluated by the percentage of transfected cells (Supplementary Figure 1). All the cell experiments were repeated in triplicates.

### Cell counting kit-8 (CCK8) assay

1×10^4^ transfected cells were seeded in a 96-well plate. After 0, 24, 48 and 72 h following the seeding, 10 μL of CCK8 reagent was added to each well and quantified using a microplate reader at 450 nm.

### Edu staining

Transfected MC3T3-E1 cells were seeded into 48-well plates. 24 h after the seeding, Edu regent (Sigma-Aldrich) was added to each well. After 2h, the cells were fixed with 4% formaldehyde for 15 min and treated with 0.5% Triton X-100 for permeabilization. Then, Apollo reaction cocktail was added to each well, followed by cell staining with Hoechst 33258.

### Cell apoptosis assay

Cell apoptosis was assessed by staining cells with Annexin V-fluorescein isothiocyanate (FITC) and propidium iodide (PI) according to the manufacturer’s instructions.

### Western blot

Total proteins were isolated from cells or calluses. Equal amounts of protein were loaded onto the wells, separated in 10% sodium dodecyl sulfate-polyacrylamide (SDS-PAGE) gel, and transferred to PVDF membranes by electrophoresis. The membranes were incubated with the primary antibodies at 4 °C overnight, then with HRP-conjugated secondary antibodies for 2 h at room temperature. The primary antibodies used are as follows: anti-PIK3R1 (1:1000, Abcam, USA, #ab86714), anti-Cyclin D1 (1:1000, Abcam, MA, USA, #ab40754), anti-Cyclin D3 (1:1,000, CST, USA, #2936), anti-Bax (1:2000, CST, USA, #2772), anti-Bcl-2 (1:2000, Abcam, MA, USA, #ab196495), anti-RunX2 (1:500, Abcam, MA, USA, #ab23981), anti-BMP2 (1:1000, Abcam, MA, USA, #ab14933), anti-GAPDH (1:10,000, Abcam, USA, #ab37168).

### qRT-PCR

Total RNAs were isolated from cells or calluses using TRIzol reagent (Invitrogen, USA). qRT-PCR was performed on a real-time PCR system. The samples were treated with RNase R (3 u/mg) for 15 min. GAPDH and U6 snRNA were selected as controls for normalizing mRNA or miRNA, respectively. The results were analyzed using the comparative Ct method (2^-ΔΔCt^). The primers used are listed in Supplementary Table 1.

### Target prediction

The open online bioinformatic tools Targetscan (http://www.targetscan.org/mamm_31/) and miRBD (http://mirdb.org/) were used to predict the mRNAs targeting miR-7212-5p. CircBase (http://www.circbase.org) was used to predict the potential circRNAs targeting miR-7223-5p.

### Luciferase reporter assay

To study the interaction of miR-7223-5p with circRNA AFF4 MC3T3-E1 cells were co-transfected with wild-type (WT) or mutated circRNA AFF4 reporter plasmids and miR-7223-5p or miR-NC. Similarly, MC3T3-E1 cells were co-transfected with either the WT or mutated FGFR3 reporter plasmids and miR-7223-5p or miR-NC. The luciferase signal was evaluated using the Dual Luciferase Reporter Assay according to the manufacturer’s instructions.

### RNA fluorescence *in situ* hybridization (FISH)

To investigate the intracellular localization circAFF4, we seeded cells into 6-well plates. After 24 h, we fixed cells by incubating them with 4% paraformaldehyde for 20 min. The cells were then incubated in a hybridization buffer with Cy3-labeled circRNA AFF4 probes at 37 °C overnight. The tissue sections were deparaffinized and permeabilized by proteinase K treatment at 37 °C for 30min. Then, the slices were incubated in the hybridization buffer with Cy3-labeled circRNA AFF4 probes and FAM-labeled miR-7223-5p at 37 °C overnight. The nuclei were counterstained with 4,6-diamidino-2-phenylindole (DAPI). The images were obtained using a confocal microscope (Nikon, Tokyo, Japan). The sequences of the probes were as follows: circRNA AFF4: 5′-CY3-GCTGCTCTTCCTCATCTC TGCTGTA CCTC-3′; miR-7223-5p: 5′-FAM-CCGA CACCTCAGCAAAGCTA-3′.

### Femoral fracture model in mice

C57BL/6 mice were purchased from Tongji Medical Laboratory Animal Center. All the animal experiments were approved by the Animal Research Committee of Tongji Medical College, Huazhong University of Science and Technology. Femoral fractures were created on mice. Briefly, a transverse osteotomy on the femur was created, and the bones were fixed with a 23-gauge needle intramedullary. Equal amounts (30 μL) of PBS, AgomiR-7223-5p, and Plasmid CircRNA AFF4 were injected into the fracture site on days 0, 4, and 7. The mice were sacrificed at a specific timepoint, and samples of calluses were harvested for WB, qRT-PCR, and microCT analysis.

### Micro-CT and radiography examination

The femoral fracture sites were scanned *ex vivo* and three-dimensional images were reconstructed using a microCT system (BRUKER, Germany). The BV, TV, BV/TV and BMD values were calculated to assess the quality of calluses.

### CircRNAs in vivo precipitation (circRIP)

CircAFF4-overexpressing MC3T3-E1 cells were fixed by incubation with 1% formaldehyde for 10 min and lysed. After centrifugation at 4 °C and 10,000 g for 10 min, 50 μL of the supernatant was retained. The remaining supernatant was incubated with streptavidin dyna-beads (M-280; Invitrogen) overnight. On the following day, the streptavidin dyna-beads-circAFF4 probes mixture was incubated with 200 μL of lysis buffer for 2 h to reverse the formaldehyde cross-linking. Finally, TRIzol (Invitrogen, USA) was used to isolated RNA from the mixture for qRT-PCR.

### Statistical analysis

All the data are shown as mean ± SD. The results were analyzed with Graph Prism 8.0 (GraphPad Software, USA). Student *t*-test or one-way analysis of variance (ANOVA) with Tukey’s post-hoc were performed accordingly. *p*<0.05 was considered statistically significant.

## Supplementary Material

Supplementary Figure 1

Supplementary Table 1
