# Supplementary material for: CircRNA AFF4 promotes osteoblast cells proliferation and inhibits apoptosis via the Mir-7223-5p/PIK3R1 axis
Source: Aging (Albany NY). 2019 Dec 17;11(24):11988–2001. doi: 10.18632/aging.102524 (PMC6949079; doi:10.18632/aging.102524)
Supplement: Supplementary Figure 1 [file aging-11-102524-s002..pdf]

## SUPPLEMENTARY FIGURE

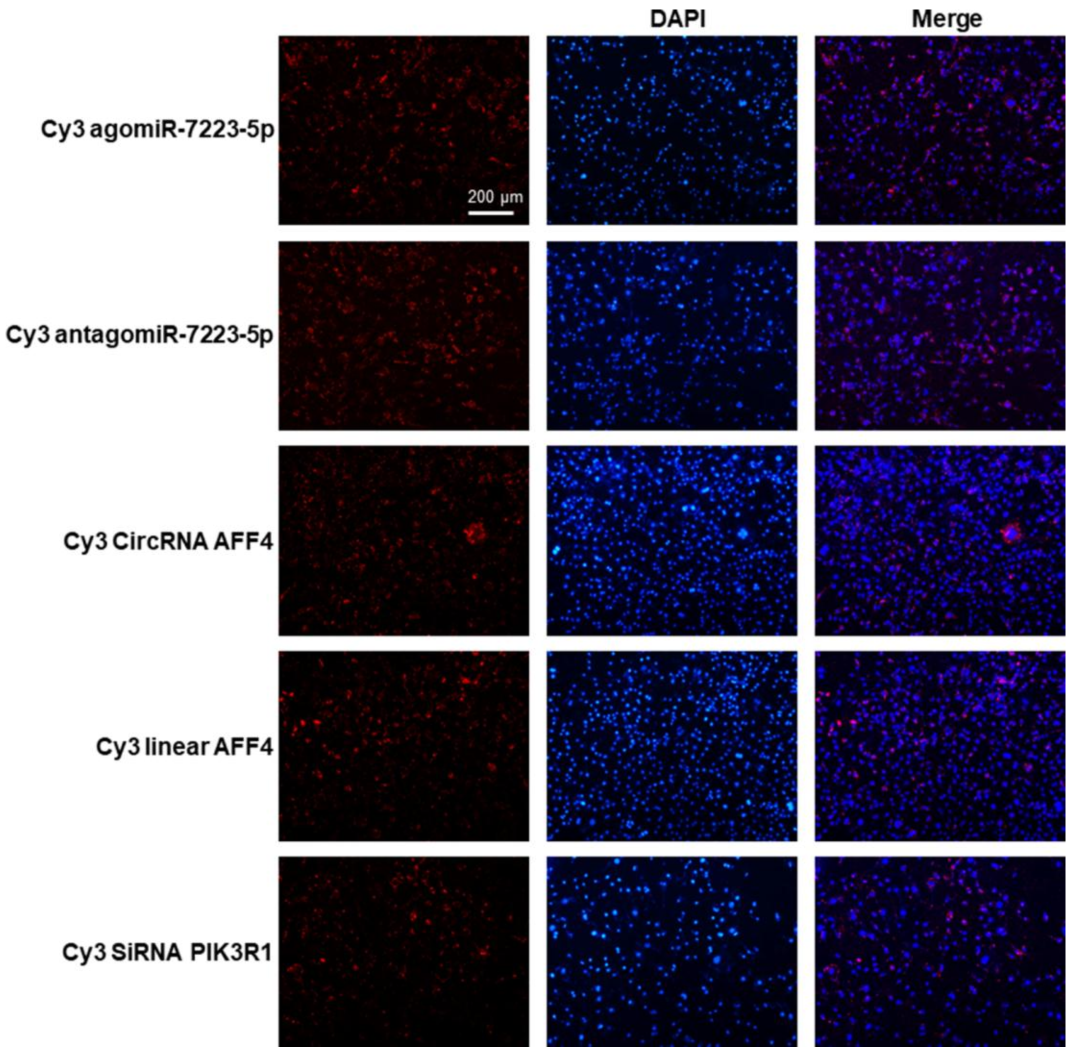


**Supplementary Figure 1. The transfection rate of RNAs.** AgomiR-7223-5p, antagomiR-7223-5p, circRNA AFF4, linear AFF4 and siRNA PIK3R1 transfecting cultured MC3T3-E1 cells as confirmed by Cy3. Scar bar=200 μm.
