# Supplementary material for: CircRNA AFF4 promotes osteoblast cells proliferation and inhibits apoptosis via the Mir-7223-5p/PIK3R1 axis
Source: Aging (Albany NY). 2019 Dec 17;11(24):11988–2001. doi: 10.18632/aging.102524 (PMC6949079; doi:10.18632/aging.102524)
Supplement: Supplementary Table 1 [file aging-11-102524-s001..pdf]

Supplementary Table

Supplementary Table 1. The primer sequence of circRNA, miRNA and mRNA.

microRNAs or gene name	Primer sequence (5' to 3')
CircRNA AFF4-Forward	GCATCGGTTTCTGGTGATGT
CircRNA AFF4-Reverse	CGGTTCATGTTGCTTAGTTG
Circ-β-actin-Forward	GACGGCCAGGTCATCACTAT
Circ-β-actin-Reverse	GAAGGTCTCAAACATGATCCAAGAA
U6-Forward	CTCGCTTCGGCAGCACA
U6-Reverse	AACGCTTCACGAATTTGCGT
PIK3R1-Forward	CCAAATGAAAAGAACGGCTATC
PIK3R1-Reverse	TACGCGACTTCAGCTTATCATG
Runx2-Forward	CGCCACCACTCACTACCACAC
Runx2-Reverse	TGGATTTAATAGCGTGCTGCC
BMP2-Forward	ATGTGAGGATTAGCAGGTCTTTG
BMP2-Reverse	TCTCTTGCAGCTGGACTTGAG
GAPDH-Forward	AGAGTGTTTCCTCGTCCCG
GAPDH-Reverse	CCGTTGAATTTGCCGTGA
